# Complement Activation-Independent Attenuation of SARS-CoV-2 Infection by C1q and C4b-Binding Protein

**DOI:** 10.3390/v15061269

**Published:** 2023-05-29

**Authors:** Nazar Beirag, Praveen M. Varghese, Martin Mayora Neto, Ahmad Al Aiyan, Haseeb A. Khan, Moneeb Qablan, Mohamed H. Shamji, Robert B. Sim, Nigel Temperton, Uday Kishore

**Affiliations:** 1 Biosciences, College of Health, Medicine and Life Sciences, Brunel University London, Uxbridge UB8 3PH, UK; nazar.beirag@brunel.ac.uk (N.B.); praveenmathewsvarghese@gmail.com (P.M.V.); 2 School of Biosciences and Technology, Vellore Institute of Technology, Vellore 632014, India; 3 Viral Pseudotype Unit, Medway School of Pharmacy, University of Kent and Greenwich, Kent ME4 4TB, UK; m.mayora-neto@kent.ac.uk (M.M.N.); n.temperton@kent.ac.uk (N.T.); 4 Department of Veterinary Medicine, United Arab Emirates University, Al Ain P.O. Box 15551, United Arab Emirates; a.alaiyan@uaeu.ac.ae (A.A.A.); m.gablan@uaeu.ac.ae (M.Q.); 5 Department of Biochemistry, College of Science, King Saud University, Riyadh 4545, Saudi Arabia; khan_haseeb@yahoo.com; 6 Immunomodulation and Tolerance Group, Department of Allergy and Clinical Immunology, National Heart and Lung Institute, Imperial College London, London SW7 2BX, UK; m.shamji99@imperial.ac.uk; 7 MRC Immunochemistry Unit, Department of Biochemistry, University of Oxford, South Parks Road, Oxford OX1 3QU, UK; edith.sim@pharm.ox.ac.uk

**Keywords:** innate immunity, complement, classical pathway, C1q, C4BP, SARS-CoV-2, COVID-19

## Abstract

The complement system is a key component of the innate immune response to viruses and proinflammatory events. Exaggerated complement activation has been attributed to the induction of a cytokine storm in severe SARS-CoV-2 infection. However, there is also an argument for the protective role of complement proteins, given their local synthesis or activation at the site of viral infection. This study investigated the complement activation-independent role of C1q and C4b-binding protein (C4BP) against SARS-CoV-2 infection. The interactions of C1q, its recombinant globular heads, and C4BP with the SARS-CoV-2 spike and receptor binding domain (RBD) were examined using direct ELISA. In addition, RT-qPCR was used to evaluate the modulatory effect of these complement proteins on the SARS-CoV-2-mediated immune response. Cell binding and luciferase-based viral entry assays were utilised to assess the effects of C1q, its recombinant globular heads, and C4BP on SARS-CoV-2 cell entry. C1q and C4BP bound directly to SARS-CoV-2 pseudotype particles via the RBD domain of the spike protein. C1q via its globular heads and C4BP were found to reduce binding as well as viral transduction of SARS-CoV-2 spike protein expressing lentiviral pseudotypes into transfected A549 cells expressing human ACE2 and TMPRSS2. Furthermore, the treatment of the SARS-CoV-2 spike, envelope, nucleoprotein, and membrane protein expressing alphaviral pseudotypes with C1q, its recombinant globular heads, or C4BP triggered a reduction in mRNA levels of proinflammatory cytokines and chemokines such as IL-1β, IL-8, IL-6, TNF-α, IFN-α, and RANTES (as well as NF-κB) in A549 cells expressing human ACE2 and TMPRSS2. In addition, C1q and C4BP treatment also reduced SARS-CoV-2 pseudotype infection-mediated NF-κB activation in A549 cells expressing human ACE2 and TMPRSS2. C1q and C4BP are synthesised primarily by hepatocytes; however, they are also produced by macrophages, and alveolar type II cells, respectively, locally at the pulmonary site. These findings support the notion that the locally produced C1q and C4BP can be protective against SARS-CoV-2 infection in a complement activation-independent manner, offering immune resistance by inhibiting virus binding to target host cells and attenuating the infection-associated inflammatory response.

## 1. Introduction

Severe acute respiratory syndrome coronavirus type 2 (SARS-CoV-2) caused the COVID-19 pandemic, resulting in approximately 472 million infection cases and over 20 million fatalities worldwide [1]. The pathogenesis of COVID-19 is mainly associated with immune response dysregulation. SARS-CoV-2 is an enveloped, positive-sense RNA virus belonging to the Coronaviridae family [2]. The virus comprises four primary structural proteins, including nucleocapsid (N), membrane spike (S), membrane (M), and a small hydrophobic membrane (E) protein, while other auxiliary proteins also facilitate viral entry and replication [2]. The S protein covers the surface of SARS-CoV-2 and contains two subunits, S1 and S2, which serve as host cell receptor-binding proteins for the virus [2]. SARS-CoV-2 infects various cell types, including alveolar macrophages and epithelial cells, by binding to the angiotensin-converting enzyme 2 (ACE2) receptor [3].

The complement system is a major protagonist in innate and acquired immunity, contributing significantly to the body’s defence against viral, bacterial, fungal, and protozoan infections [4]. It comprises more than 30 proteins that can trigger an enzymatic cascade in response to various stimuli, including pathogen-associated molecular patterns (PAMPs) and aberrant or injured host cells [4]. The complement system is activated via three pathways (classical, lectin, and alternative), all of which converge at one point: the cleavage of complement component 3 (C3) [4]. The classical pathway is mainly activated by the binding of C1q to immune complexes that include IgG and IgM, while the lectin pathway is activated when mannan-binding lectin (MBL) recognizes non-self-carbohydrate structures [4]. On the other hand, the alternative pathway is activated when C3 spontaneously undergoes hydrolysis to generate C3b, which then interacts with various proteins, lipids, and carbohydrates on the pathogen surface [4]. Briefly, activation of either the classical or lectin pathway leads to the breakdown of C4 and C2, resulting in the formation of C3 convertase (C4b2b), which cleaves C3 to produce C3b [5]. In the alternative pathway, hydrolysis of the internal thioester bond in C3 produces C3(H_2_O), which binds to factor B, leading to its cleavage by factor D into Bb [5]. This generates C3(H_2_O) Bb, similar to the classical pathway [5].

Studies involving coronaviruses such as MERS-CoV have implicated the complement system in causing hyperinflammation in animal models [6]. Similarly, SARS-CoV was found to interact with MBL and activate the lectin pathway [7].

Recently, evidence of complement activation in COVID-19, including elevated serum concentrations of C5a and C5b-9, increased leukocyte CD11b expression (which can result from C5aR1 activation), and post-mortem immunochemistry, has been linked to disease severity [8]. SARS-CoV-2 has been shown to activate the complement via all three pathways [9]. Furthermore, dysregulation of the classical pathway has been associated with the pathogenesis of lung injury in SARS-CoV-2 infection, with reduced serum levels of the key classical pathway proteins, including C1q and C4BP, in patients suffering from severe COVID-19 [10,11]. Additionally, SARS-CoV-2 exploits weaknesses in the interferon system in these patients and replicates unchecked [8], which in turn, is believed to trigger a complement-mediated hyperinflammation when the virus reaches a critical mass.

C1q is the first subcomponent of the classical pathway that regulates various homeostatic processes, including removal of immune complexes, pathogens, and apoptotic cells [4]. C1q has been reported to protect the host from viral infections, as in the case of West Nile virus (WNV) infection that may result in respiratory paralysis; C1q is required for the normal humoral response to WNV [12]. Another example is the binding of C1q to antibody-bound respiratory syncytial virus (RSV) that leads to activation of the classical pathway [13]. C1q has also been implicated in regulating anti-viral antibody-mediated effector mechanism in influenza viral infection [14]. Another complement key protein is C4b-binding protein (C4BP), a crucial fluid phase inhibitor of the classical and lectin pathways [4]. C4BP inhibits the generation of C3 and C5 convertases and acts as a cofactor for factor I [4]. Variants in the gene encoding C4BP α chain are considered risk factors for morbidity and mortality in SARS-CoV-2 infection [15].

While complement proteins are mainly believed to be synthesized by hepatocytes and circulate in the serum, studies have shown that C1q can be produced locally in the lungs by macrophages and immature dendritic cells; C4BP can also be produced locally in the lungs by alveolar type II cells. This suggests its crucial role in protecting the pulmonary tissue during initial stages of infection [5,16,17,18]. These locally produced C1q and C4BP, independent of other complement proteins or complement activation, were found to independently act as soluble pattern recognition receptors that interact with influenza A virus (IAV) surface proteins, and thus modulate viral entry and subsequent viral replication in a subtype-dependent manner [5,19].

Despite several studies examining the role of classical and alternative pathways in the pathogenesis of SARS-CoV-2 infection, the immune functions of C1q and C4BP in the infection, independent of the complement activation, have not been evaluated. Here, we used purified human C1q, its recombinant globular heads (ghA, ghB, and ghC), and C4BP proteins to study their likely protective or pathogenic role against SARS-CoV-2 infection in a complement activation-independent manner. Furthermore, we investigated the interaction of C1q and C4BP with SARS-CoV-2 S and RBD proteins and their entry inhibition potential against infection by SARS-CoV-2 pseudotype viral particles in A549 cells expressing human ACE2 and TMPRSS2 receptors/co-receptors. Here, we demonstrate that C1q and C4BP can inhibit SARS-CoV-2 pseudotype cell entry independent of complement activation or the antibody response. In addition, the proinflammatory response triggered by SARS-CoV-2 was downregulated by C1q and C4BP treatment.

## 2. Materials and Methods

### 2.1. Purification of Native Human C1q

Human C1q was purified, as previously described [20]. Briefly, fresh frozen human plasma (100 mL) was centrifuged at 5000× *g* for 10 min, then passed through a Whatman filter paper (GE Healthcare, Hatfield, UK) to remove lipids. The plasma was then incubated with IgG-Sepharose (GE Healthcare, Hatfield, UK) for 2 h at room temperature (RT). The column was washed with a wash buffer (10 mM HEPES) and C1q was eluted using the elution buffer (100 mM CAPS, 1 M NaCl, 0.5 mM EDTA, pH 11). The eluted C1q was applied to a Hi-Trap Protein G column to remove residual IgG, and the flowthrough was collected. Finally, the purified C1q was dialysed against a 0.1 M HEPES buffer, pH 7.5, quantified (yield ~ 2 mg), and examined via SDS-PAGE (Supplementary Figure S1A).

### 2.2. Purification of Recombinant ghA, ghB, and ghC Modules of Human C1q

Recombinant forms of human C1q globular head regions of A (ghA), B (ghB), and C (ghC) chains, linked to maltose-binding protein (MBP), were produced in *E. coli* BL21 cells [21]. In total, 12.5 mL of the primary culture of the cells expressing the proteins was inoculated into a 500 mL LB medium supplemented with 100 μg/mL of ampicillin. The mixture was then placed on a shaker and incubated at 37 °C for 3 h until the OD_600_ reached 0.6. Subsequently, the culture was induced with 0.4 mM of IPTG, and then incubated on a shaker at 37 °C for another 3 h. Following a centrifugation at 13,800× *g* for 10 min, the bacterial cell pellet was lysed in 25 mL of a lysis buffer (20 mM Tris-HCl, pH 8.0, 0.5 M NaCl, 1 mM EGTA, pH 7.5, 1 mM EDTA, pH 7.5, 5% *v*/*v* glycerol, 0.2% *v*/*v* Tween 20, 0.1 mM PMSF, and 50 µg/mL lysozyme) for 30 min at 4 °C. The lysate was subjected to sonication for 12 cycles at 60 Hz for 30 sec with an interval of 2 min. Next, the lysate was centrifuged at 15,000× *g* for 30 min at 4 °C. The supernatant was mixed with 125 mL of buffer I (20 mM Tris-HCl, pH 8.0, 100 mM NaCl, 1 mM EDTA, pH 7.5, 0.2% *v*/*v* Tween 20, and 5% *v*/*v* glycerol). The five-fold diluted supernatant was loaded onto an amylose resin (5 mL) column (New England Biolabs). The column was then washed with 150 mL of buffer I, followed by 250 mL of buffer II (buffer I without Tween 20). The fusion protein was eluted using 100 mL of buffer II containing 100 mM maltose. The protein concentration was determined by measuring A_280_ before analysing the purified proteins via SDS-PAGE. The peak fractions were further passed through Pierce™ High-Capacity Endotoxin Removal Resin (Qiagen, Hilden, Germany) to eliminate the lipopolysaccharide (LPS). Finally, the endotoxin levels in the purified protein samples were assessed using the QCL-1000 Limulus amebocyte lysate system (Lonza, Basel, Switzerland). The recombinant proteins were found to have endotoxin levels of ~4 pg/μg. The purified proteins were quantified (yield ~ 1.5 mg of each globular head) and examined via SDS-PAGE (Supplementary Figure S1B).

### 2.3. Purification of Native Human C4BP

C4BP was obtained from the neutral euglobulin precipitation and affinity chromatography, utilizing C4c-Sepharose, as previously described [22]. Fresh frozen pooled human plasma (100 mL) was adjusted to pH 7.5 by adding 0.15 N HCl. The ionic strength was reduced by adding 4 volumes of cold distilled water. The mixture was stirred at pH 7.5 for 30 min at 4 °C. The euglobulin fraction, which contained over 95% of the C4BP activity, was obtained by centrifugation for 45 min at 14,000× *g*. The resulting precipitate was washed off with a VB^++^ solution (5 mM sodium 5,5-diethyl barbiturate, 142 mM NaCl, 0.025% NaN_3_, pH 7.5, 0.15 mM CaCl_2_, and 0.5 mM MgCl_2_). It was then re-dissolved in 40 mL of an isotonic VB^++^ solution by gentle stirring for several hours at 4 °C. The solution was clarified by re-centrifugation, and the precipitation process at pH 7.5 and an ionic strength of 0.03 mol/L was repeated. The final precipitate was dissolved in 30 mL of a cold VB^++^ solution and clarified by centrifugation. The C4c-Sepharose column was initially equilibrated and washed in a 5 mM EDTA/25 mM potassium phosphate buffer, pH 7.0. The protein was then eluted by a linear gradient of NaCl (0 to 2 M); C4BP was eluted at 0.8 M NaCl. The purified C4BP was quantified (yield ~ 2.5 mg) and assessed via SDS-PAGE (Supplementary Figure S1C).

### 2.4. Direct-Binding ELISA

To examine the binding of SARS-CoV-2 S or RBD proteins to the immobilised C1q or C4BP, various concentrations of C1q or C4BP (1, 0.5, 0.125, and 0 pmol/well) were immobilised on polystyrene microtiter wells (Sigma-Aldrich, St. Louis, MO, USA) using a carbonate/bicarbonate (CBC) buffer, pH 9.6, (Sigma-Aldrich) overnight at 4 °C. As a negative control, wells were coated with 15 pmol/well BSA. The following day, the wells were washed 3 times with a PBST buffer (PBS + 0.05% Tween 20) (Fisher Scientific, Hampton, NH, USA) to eliminate any non-specifically bound proteins. Following the washes, 2% *w*/*v* BSA in PBS (Fisher Scientific) was used to block the wells for 2 h at 37 °C and then they were washed three times with PBST to remove any excess of BSA. A constant dose of 3 pmol/well recombinant SARS-CoV-2 S protein (RP-87680, Invitrogen, Waltham, MA, USA) or 30 pmol/well recombinant SARS-CoV-2 RBD protein (40592-V08H, Sino-Biological, Beijing, China) was added into the respective wells where C1q or C4BP were immobilised.

In parallel experiments, fixed concentrations of C1q or C4BP (1, 0.5, 1.25, and 0 pmol/well) were added to immobilised SARS-CoV-2 S (3 pmol/well) or RBD (30 pmol/well) coated wells. The plate was washed three times with PBST and blocked with 2% *w*/*v* BSA in PBS for 2 h at 37 °C, and washed three times with PBST again. BSA was used as a negative control. The binding of SARS-CoV-2 S or RBD to immobilised C1q or C4BP binding was detected using the polyclonal rabbit anti-SARS-CoV-2 spike (NR-52947, BEI-Resources). The binding of C1q or C4BP to the immobilised SARS-CoV-2 spike or RBD protein was detected using the corresponding primary antibodies, rabbit anti-human C1q or rabbit anti-human C4BP (both produced in the MRC immunochemistry unit, Oxford, UK) polyclonal antibodies, respectively. The primary antibodies were used at a concentration of 1:5000 and incubated for 1 h at 37 °C. The wells were washed 3 times using PBST to remove any unbound antibodies. Goat anti-rabbit IgG conjugated to horseradish peroxidase (HRP) (1:5000 dilution) (Promega, Madison, WI, USA) was used as a secondary antibody and incubated for 1 h at 37 °C. Finally, 100 µL/well of a 3,3’,5,5’-tetramethylbenzidine (TMB) substrate set (Biolegend, San Diego, CA, USA) was used to detect the binding; the reaction was stopped with 100 µL/well of 1 M of H_2_SO_4_ (Sigma-Aldrich). The plate was read using an iMark™ microplate absorbance reader (BioRad, Hercules, CA, USA) at 450 nm.

### 2.5. Cell Culture

A549 lung epithelial cells were cultured in Dulbecco’s Modified Eagle’s Medium (DMEM) with Glutamax (Gibco, Cambridge, UK) supplemented with 100 U/mL penicillin (Gibco), 100 µg/mL streptomycin (Gibco), and 10% *v*/*v* foetal bovine serum (FBS) (Gibco). The cells were cultured at 37 °C and 5% *v*/*v* CO_2_ until they were 70% confluent. Then, using Promega FuGENE^TM^ HD Transfection Reagent, the cells were transiently co-transfected with a plasmid expressing human ACE2 (pCDNA3.1+-ACE2) and another expressing TMPRSS2 (pCAGGS-TMPRSS2). The next day, cells were cultured with hygromycin and puromycin (Thermo Fisher Scientific, Waltham, MA, USA) to select A549 cells co-expressing human ACE2 and TMPRSSS2 (A549-hACE2+TMPRSS2 cells). Anti-hACE2 (Sino Biological Inc., Beijing, China, Cat: 80031-RP01) and anti-TMPRSS2 (Sino Biological Inc., Cat: 204314-T08) antibodies were used to evaluate hACE2 and TMPRSS2 expression, respectively, using western blotting (Supplementary Figure S2A,B).

### 2.6. Viral Cell Entry Assay

#### 2.6.1. Preparation of SARS-CoV-2 Pseudotyped Lentiviral Particles

Pseudotyped lentiviral particles were generated as previously described [23]. Briefly, human embryonic kidney (HEK) 293T/17 cells were cultured up to 70–90% confluence at 37 °C and 5% *v*/*v* CO_2_ in DMEM growth media with 4.5 g/L glucose (Pan-Biotech, Aidenbach, Germany) supplemented with 10% FBS (Pan-Biotech) and 1% penicillin/streptomycin (Pan Biotech). In addition, pCAGGS-SARS-CoV-2 spike plasmids (CFAR, Catalog number: 100976), the lentiviral vector expressing firefly luciferase pCSFLW [23], and the second-generation lentiviral packaging construct p8.91 (expressing *gag*, *pol,* and *rev*) [23] were utilised to transfect the cells. Supernatants containing the pseudotype viral particles were harvested using a 3 mL sterile syringe and subsequently filtered into Falcon tubes via a syringe-driven 0.45 µm filter. All filtered supernatants were stored at −80 °C.

#### 2.6.2. Treatment of SARS-CoV-2 Pseudotyped Lentiviral Particles

SARS-CoV-2 lentiviral pseudotyped particles were used for a luciferase reporter-based cell entry assay. The pseudotyped particles were pre-incubated with 40 µmol/mL C1q or C4BP, and 3.33 × 10^2^ µmol/mL of gh modules (ghA, ghB, or ghC), for 2 h at RT. The pre-incubated mixture was then used to challenge A549-hACE2 + TMPRSS2 cells. SARS-CoV-2 lentiviral pseudoparticles + A549-hACE2 + TMPRSS2 cells were considered untreated control cells for C1q and C4BP. SARS-CoV-2 lentiviral pseudoparticles + MBP + A549-h ACE2 + TMPRSS2 cells were regarded as untreated control cells for the ghs.

### 2.7. Luciferase Reporter Assay

A luciferase reporter assay was used to assess if C1q, ghA, ghB, ghC, or C4BP treatment could affect SARS-CoV-2 pseudotype particle cell entry. Briefly, A549-hACE2 + TMPRSS2 cells (20,000 cells/well) were seeded in a 96-well plate and incubated in complete growth media overnight at 37 °C. The cells were then challenged with C1q, ghA, ghB, ghC, MBP or C4BP treated SARS-CoV-2 lentiviral pseudoparticles in incomplete growth medium–DMEM with Glutamax (Gibco) supplemented with 100 U/mL penicillin (Gibco) and 100 μg/mL streptomycin (Gibco), and incubated at 37 °C for 24 h. Next, the cells were washed twice in PBS, and a complete fresh medium was added and incubated for another 48 h at 37 °C. Subsequently, luciferase activity (RLU) was measured using the ONE-Glo^TM^ Luciferase Assay System (Promega) and read on the Clariostar Plus Microplate Reader (BMG Labtech, Cary, NC, USA).

### 2.8. NF-κB Activity Assay

A luciferase-based reporter assay was used to measure NF-κB activation to determine how C1q and C4BP affected NF-κB activity during SARS-CoV-2 infection. The assay is based on a plasmid that consists of multiple copies of the NF-κB consensus sequences fused to a TATA-like promoter region of the herpes simplex virus thymidine kinase (HSV-TK) promoter. This vector has been engineered to directly measure the NF-κB pathway by assessing the transcription factor’s binding to the κ-enhancer. The reporter gene is activated, and transcription is induced once endogenous NF-κB binds to the κ-enhancer region. A549-hACE2+TMPRSS2 cells were transfected with the pNF-κB-LUC plasmid (T 631904; Clonetech, Fitchburg, WI, USA) using the Promega FuGENE^TM^ HD Transfection Reagent and incubated at 37 °C in a complete growth medium for 48 h. Post transfection, the cells (20,000 cells/well) were plated in a 96-well plate and left overnight in a complete growth medium at 37 °C. This was followed by challenging the cells with SARS-CoV-2 S protein (S protein 2.7 × 10^3^ µmol/mL) pre-treated with 40 µmol/mL C1q or C4BP for 2 h at RT and they were incubated for 24 h at 37 °C in an incomplete growth medium. A549-hACE2+TMPRSS2 cells + SARS-CoV-2 S protein were used as a control. The luciferase activity corresponding to NF-κB activation was measured as mentioned above.

### 2.9. Cell Binding Assay

The effect of C1q or C4BP treatment on SARS-CoV-2 pseudotype binding to transfected A549 cells was evaluated using a cell-binding assay. Briefly, A549-hACE2+TMPRSS2 cells (20,000 cells/well) were seeded in a 96-well plate in a growth medium and left overnight at 37 °C. The next day, cells were challenged with SARS-CoV-2 lentiviral pseudoparticles treated with C1q, ghA, ghB, ghC, or C4BP and incubated in an incomplete growth medium for 2 h at 37 °C. This was followed by washing the plate three times with PBS; cells were then fixed for 1 min using 1% *v*/*v* paraformaldehyde (PFA) at RT. After washing three times with PBS, cells were incubated with rabbit anti-SARS-CoV-2 spike (1:200) polyclonal antibodies for 1 h at 37 °C and washed. Finally, the wells were probed with the Alexa Fluor 488 conjugated goat anti-rabbit antibody (1:200) (Abcam, Cambridge, UK) for 1 h at RT. The plate was read using a Clariostar Plus Microplate Reader (BMG Labtech, Cary, NC, USA).

### 2.10. Modulation of SARS-CoV-2 Pseudoparticle-Induced Infection by C1q or C4BP

SARS-CoV-2 alphaviral pseudoparticles containing the four structural proteins, S, E, M, and N (Ha-CoV-2 Luc; Virongy, Manassas, VA, USA), pre-incubated with 40 µmol/mL C1q or C4BP for 2 h at RT, were used to challenge A549-hACE2+TMPRSS2 cells. Cytokine/chemokine gene expression was analysed via RT-qPCR (see below). SARS-CoV-2 alphaviral pseudoparticles+A549-hACE2+TMPRSS2 cells alone were used as a control.

#### Quantitative qRT-PCR Analysis

RT-qPCR was used to assess if C1q or C4BP treatment could impact proinflammatory gene expression levels in cells challenged with the SARS-CoV-2 pseudotype. Briefly, A549-hACE2 + TMPRSS2 cells (0.5 × 10^6^) were seeded in a 12-well plate and incubated overnight at 37 °C and 5% *v*/*v* CO_2_ in a complete growth medium. SARS-CoV-2 alphaviral pseudoparticles pre-treated with C1q or C4BP (as described above) were added to A549-hACE2 + TMPRSS2 cells and incubated for 6 and 12 h at 37 °C in incomplete growth media, respectively. The cells were washed with PBS and pelleted. The total RNA was extracted using the GenElute Mammalian Total RNA Purification Kit (Sigma-Aldrich). NanoDrop 2000/2000c (ThermoFisher) was used to measure the amount of RNA at A260 nm after DNA contaminants were removed using DNase I (Sigma-Aldrich). The purity of the RNA was assessed using the A260/A280 ratio. In total, 2 µg of total RNA was utilised to synthesise cDNA using the High-Capacity RNA to cDNA Kit (Applied Biosystems, Waltham, MA, USA). The primer BLAST software generated the primer sequences (Basic Local Alignment Search Tool) (Table 1). The qRT-PCR was run using Step One Plus (Applied Biosciences). Each qPCR (in triplicate) contained 500 ng of cDNA, 75 nM of forward and reverse primers, and 5 µL of Power SYBR Green Master Mix (Applied Biosystems). qPCR samples were run at 50 °C and 95 °C for 2 and 10 min, followed by running the amplification template for 40 cycles, each cycle involving 15 s at 95 °C and 1 min at 60 °C, respectively. Additionally, 18S rRNA was used as an endogenous control to normalise gene expression.

### 2.11. Statistical Analysis

GraphPad Prism 9.0 was used to create the graphs. The statistical significance between the treated and untreated conditions was considered, as shown in the figure legends. Error bars indicate SD or SEM, as stated in the figure legends.

## 3. Results

### 3.1. Human C1q and C4BP Bind to SARS-CoV-2 Spike and RBD Proteins

**Figure 1:**
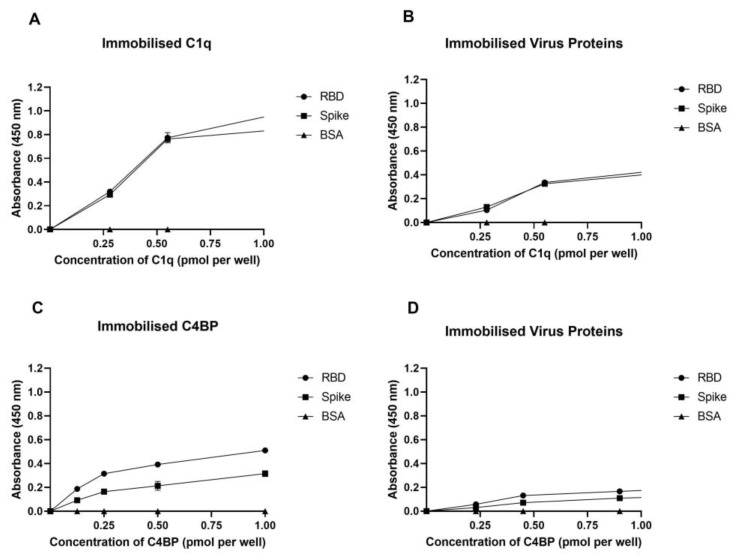
SARS-CoV-2 directly interacts with C1q and C4BP. The decreasing concentration of immobilized C1q (1, 0.5, 0.125, and 0 pmol/well) (**A**,**C**) or constant concentrations of viral proteins (spike 3 pmol/well or RBD 30 pmol/well) (**B**,**D**) were coated in a 96-well plate using a carbonate–bicarbonate (CBC) buffer, pH 9.6, at 4 °C overnight. A constant concentration of viral proteins (1 pmol/well) (**A**) or a decreasing amount of C1q/C4BP (1, 0.5, 0.125, and 0 pmol/well) (**B**) was added to the corresponding wells, and incubated at 37 °C for 2 h. After washing step, the wells were probed with primary antibodies (1:5000; 100 µL/well), i.e., rabbit anti-SARS-CoV-2 spike or rabbit anti-human C1q/C4BP. BSA was used as a negative control. The data are presented as a mean of three independent experiments carried out in triplicates ± SEM.

A direct ELISA was used to determine the ability of immobilized native C1q to bind to SARS-CoV-2 S and RBD proteins (Figure 1A) and vice versa (Figure 1B). The interaction between immobilized C1q and SARS-CoV-2 S and RBD proteins was found to be dose-dependent when probed with the anti-SARS-CoV-2 S protein polyclonal antibody. Immobilized SARS-CoV-2 spike protein or RBD also exhibited dose-dependent binding to C1q when probed with the rabbit anti-human C1q polyclonal antibody.

Similarly, the ability of immobilized native C4BP to bind to SARS-CoV-2 S and RBD proteins (Figure 1C) and vice versa (Figure 1D) was examined using a direct ELISA. Immobilized C4BP bound SARS-CoV-2 S as well as RBD proteins in a dose-dependent manner when tested with polyclonal anti-SARS-CoV-2 S protein. When probed with rabbit anti-human C4BP, the immobilized SARS-CoV-2 S or RBD similarly bound C4BP in a dose-dependent manner; however, the binding was weaker compared to C1q. BSA protein was used as a negative control protein.

### 3.2. Human C1q, Recombinant Globular Head Modules, and C4BP Inhibit SARS-CoV-2 Pseudoparticle Transduction

**Figure 2:**
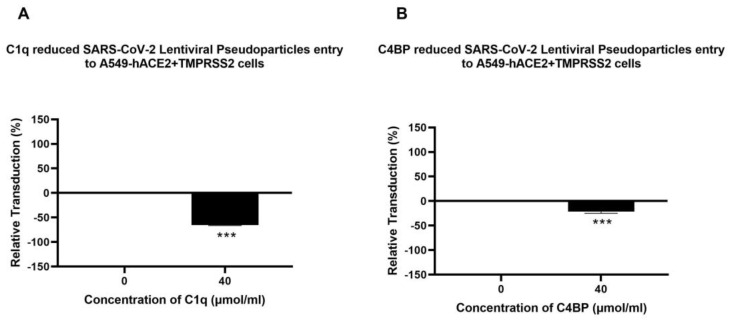
C1q (**A**) and C4BP (**B**) attenuate SARS-CoV-2 pseudoparticle entry into A549-hACE2+TMPRSS2 cells. Luciferase reporter activity of A549-hACE2+TMPRSS2 cells transduced with either treated or untreated SARS-CoV-2 lentiviral pseudoparticles pre-treated with C1q or C4BP (40 μmol/mL) was utilised to assess if the treatment by complement proteins interfered with the lentiviral pseudoparticles’ ability to enter the cells. The background was subtracted from all data points. The data obtained were normalised with 0% luciferase activity defined as the mean of the relative luminescence units recorded from the control sample (A549-hACE2+TMPRSS2 cells + SARS-CoV-2 lentiviral pseudoparticles). Pseudoparticles pre-treated with C1q and C4BP, blocked viral transduction. Data are shown as the normalized mean of three independent experiments carried out in triplicates ± SEM. Significance was determined using the two-way ANOVA test (*** *p* < 0.001) (*n* = 3).

A luciferase reporter assay was used to evaluate if C1q, ghA, ghB, ghC, and C4BP could affect SARS-CoV-2 infectivity. SARS-CoV-2 lentiviral pseudoparticles treated with C1q, ghA, ghB, ghC, or C4BP reduced viral transduction in A549-hACE2+TMPRSS2 cells with respect to their respective controls. A549-hACE2+TMPRSS2 cells, challenged with SARS-CoV-2 lentiviral pseudoparticles that were pre-treated with C1q or C4BP, reduced viral infection by ~60% (Figure 2A) and ~17% (Figure 2B), respectively, compared to the control (A549-hACE2+TMPRSS2 cells + SARS-CoV-2 pseudoparticles). Thus, C1q treatment can significantly inhibit SARS-CoV-2 viral infection in a complement-independent manner; the inhibition was not comparably pronounced for C4BP. No statistically significant difference was observed in transduction efficiency between A549 cells challenged with the SARS-CoV-2 pseudotype and the control group of native A549 cells (Supplementary Figure S3A).

**Figure 3:**
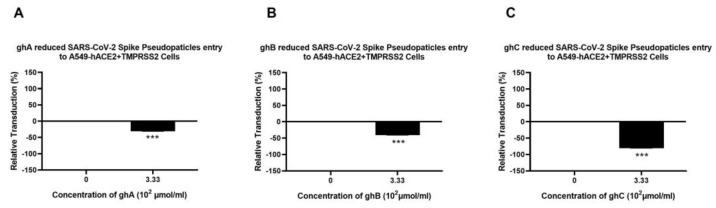
Recombinant ghA, ghB, and ghC modules of human C1q block SARS-CoV-2 pseudoparticle entry into A549-hACE2+TMPRSS2 cells. SARS-CoV-2 pseudoparticles were pre-treated with ghA (**A**), ghB (**B**), or ghC (**C**) (3.33 × 10^2^ μmol/mL) to determine if the recombinant modules of human C1q interfered with the ability of the pseudoparticles to enter the target cells. Luciferase reporter activity in A549-hACE2+TMPRSS2 cells that were transduced with pseudoparticles (and pre-treated with ghA, ghB, or ghC) was used. The background was subtracted from all data points. The data obtained were normalised with 0% luciferase activity defined as the mean of the relative luminescence units recorded from the control sample (A549-hACE2+TMPRSS2 cells + MBP + SARS-CoV-2 pseudoparticles). Data are shown as the normalized mean of three independent experiments carried out in triplicates ± SEM. Significance was determined using the two-way ANOVA test (*** *p* < 0.001) (*n* = 3).

Recombinant globular heads of C1q (ghA, ghB, and ghC) were used to test their ability to modulate SARS-CoV-2 pseudoparticle infectivity. SARS-CoV-2 lentiviral pseudoparticles, pre-treated with ghA, ghB, or ghC, caused a significant reduction in viral transduction, i.e., ~20% (Figure 3A), ~30% (Figure 3B), and ~60% (Figure 3C), respectively, when compared to the control (A549-hACE2+TMPRSS2 cells + SARS-CoV-2 lentiviral pseudoparticles +MBP). No significant difference was found between A549-hACE2+TMPRSS2 cells treated with the SARS-CoV-2 pseudotype + MBP and A549-hACE2+TMPRSS2 cells treated with the SARS-CoV-2 pseudotype alone (Supplementary Figure S4A), suggesting that the observed reduction in transduction efficiency was due to the C1q globular head modules and not due to the MBP fusion partner.

### 3.3. Human C1q, Recombinant Globular Head Modules, and C4BP Inhibit SARS-CoV-2 Pseudoparticle Binding to ACE2- and TMPRSS2-Expressing A549 Cells

**Figure 4:**
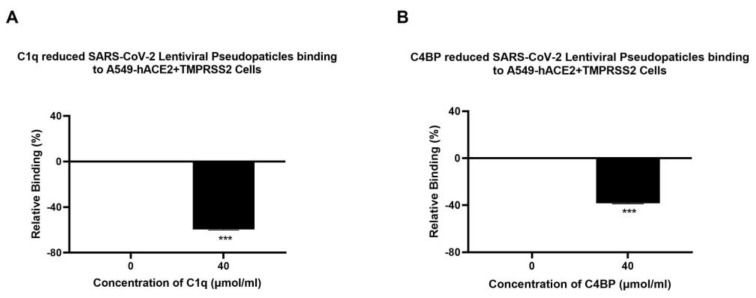
Binding of C1q (**A**) or C4BP treated (**B**) SARS-CoV-2 pseudoparticles to A549-hACE2+TMPRSS2 cells. SARS-CoV-2 lentiviral pseudoparticles were used to transduce A549-hACE2+TMPRSS2 cells, which were pre-incubated with C1q or C4BP (40 µmol/mL). The wells were probed with rabbit anti-SARS-CoV-2 spike (1:200) polyclonal antibodies after being washed and fixed with 1% *v*/*v* paraformaldehyde for 1 min. The data obtained were normalised with 0% fluorescence defined as the mean of the relative fluorescence units recorded from the control sample (A549-hACE2+TMPRSS2 cells + SARS-CoV-2 lentiviral pseudoparticles). Three independent experiments were carried out in triplicates, and error bars are expressed as ± SEM. Significance was determined using the two-way ANOVA test (*** *p* < 0.001) (*n* = 3).

A cell binding assay was carried out to assess whether C1q, ghA, ghB, ghC, and C4BP interfered with SARS-CoV-2 binding to lung epithelial-like cells. A549-hACE2+ TMPRSS2 cells were challenged with SARS-CoV-2 lentiviral pseudoparticles that were treated with either C1q, ghA, ghB, ghC, or C4BP. It showed reduced viral binding to the cells compared to their respective untreated controls. SARS-CoV-2 pseudoparticles, pre-treated with C1q or C4BP, decreased viral binding by ~65% (Figure 4A) and ~37% (Figure 4B), respectively, as compared to the control (A549-hACE2+TMPRSS2 cells + SARS-CoV-2 pseudoparticles). No statistically significant difference was observed in binding efficiency between A549 cells challenged with the SARS-CoV-2 pseudotype and the control group of native A549 cells (Supplementary Figure S3B).

**Figure 5:**
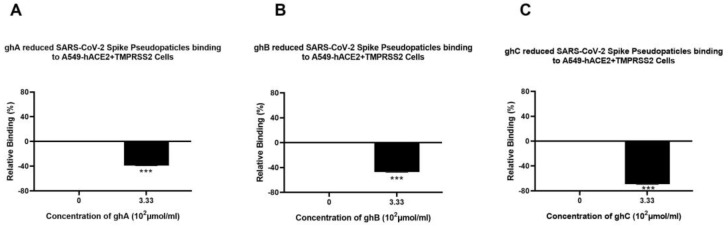
Binding of recombinant ghA- (**A**), ghB- (**B**), or ghC-treated (**C**) SARS-CoV-2 lentivirus pseudoparticles to A549-hACE2+TMPRSS2 cells. A549-hACE2+TMPRSS2 cells were transduced with SARS-CoV-2 pseudoparticles following pre-incubation with/without ghA, ghB, or ghC (3.33 × 10^2^ μmol/mL). After removing unbound protein and viral particles, the wells were fixed with 1% *v*/*v* paraformaldehyde for 1 min and probed with rabbit anti-SARS-CoV-2 spike (1:200) polyclonal antibodies. The data obtained were normalised with 0% fluorescence defined as the mean of the relative fluorescence units recorded from the control sample (cells + MBP + pseudoparticles). Three independent experiments were carried out in triplicates, and error bars express ± SEM. Significance was determined using the two-way ANOVA test (*** *p* < 0.001) (*n* = 3).

Pseudoparticles, pre-treated with ghA, ghB, or ghC, showed a reduction in viral binding of ~38% (Figure 5A), ~45% (Figure 5B), and ~70% (Figure 5C), respectively, compared to the control (A549-hACE2+TMPRSS2 cells + SARS-CoV-2 pseudoparticles + MBP). These findings suggest that C1q and C4BP inhibit SARS-CoV-2 pseudoparticle binding and entry into the target cell in a complement activation-independent manner. No significant difference was found between the group of A549-hACE2+TMPRSS2 cells treated with the SARS-CoV-2 pseudotype + MBP and the control group of A549-hACE2+TMPRSS2 cells treated with the SARS-CoV-2 pseudotype alone (Supplementary Figure S4B), suggesting that the inhibition of binding is specifically driven by the globular heads.

### 3.4. C1q and C4BP Attenuate Inflammatory Response in SARS-CoV-2 Pseudoparticles Challenged A549-hACE2+TMPRSS2 Cells

**Figure 6:**
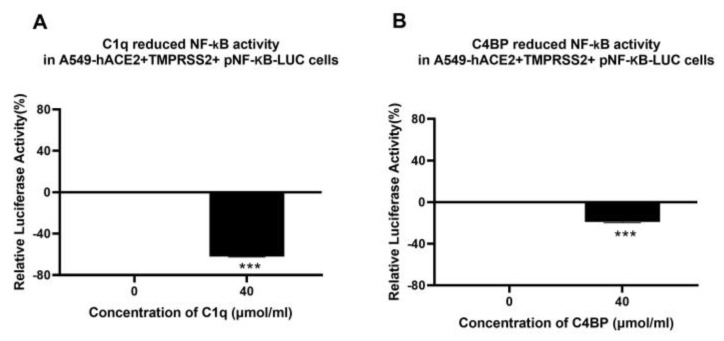
C1q and C4BP inhibit NF-κB activation in SARS-CoV-2 spike-challenged A549-hACE2-TMPRSS2 cells. A549-hACE2+TMPRSS2 cells transfected with pNF-κB-LUC were challenged with SARS-CoV-2 spike protein (1.4 µmol/mL) was pre-treated with C1q (**A**) or C4BP (**B**) (40 µmol/mL). The cells were incubated for 24 h and examined for luciferase reporter activity. The background was subtracted from all data points. The data obtained were normalised with 0% luciferase activity defined as the mean of the relative luminescence units recorded from the control sample (A549-hACE2 + TMPRSS2 cells + SARS-CoV-2 spike protein). Data are shown as the normalized mean of three independent experiments carried out in triplicates ± SEM. Significance was determined using the two-way ANOVA test (*** *p* < 0.001) (*n* = 3).

The effect of C1q or C4BP on NF-κB activation in lung epithelial-like A549 cells challenged with SARS-CoV-2 pseudoparticles was assessed using a luciferase reporter assay. The NF-κB pathway is frequently associated with a proinflammatory signal and responses. A549-hACE2+TMPRSS2 cells, challenged with SARS-CoV-2 S protein that was pre-treated with C1q, showed ~65% reduction in NF-κB activation compared to the untreated control (A549-hACE2+TMPRSS2 cells + SARS-CoV-2 spike protein) (Figure 6A). A ~17% decrease in NF-κB activation was observed in A549-hACE2+ TMPRSS2 cells challenged with SARS-CoV-2 spike protein pre-treated with C4BP with respect to the control (Figure 6B). These findings suggest that C1q, and to some extent C4BP, negate the SARS-CoV-2-induced inflammatory response by reducing NF-κB activation. 

**Figure 7:**
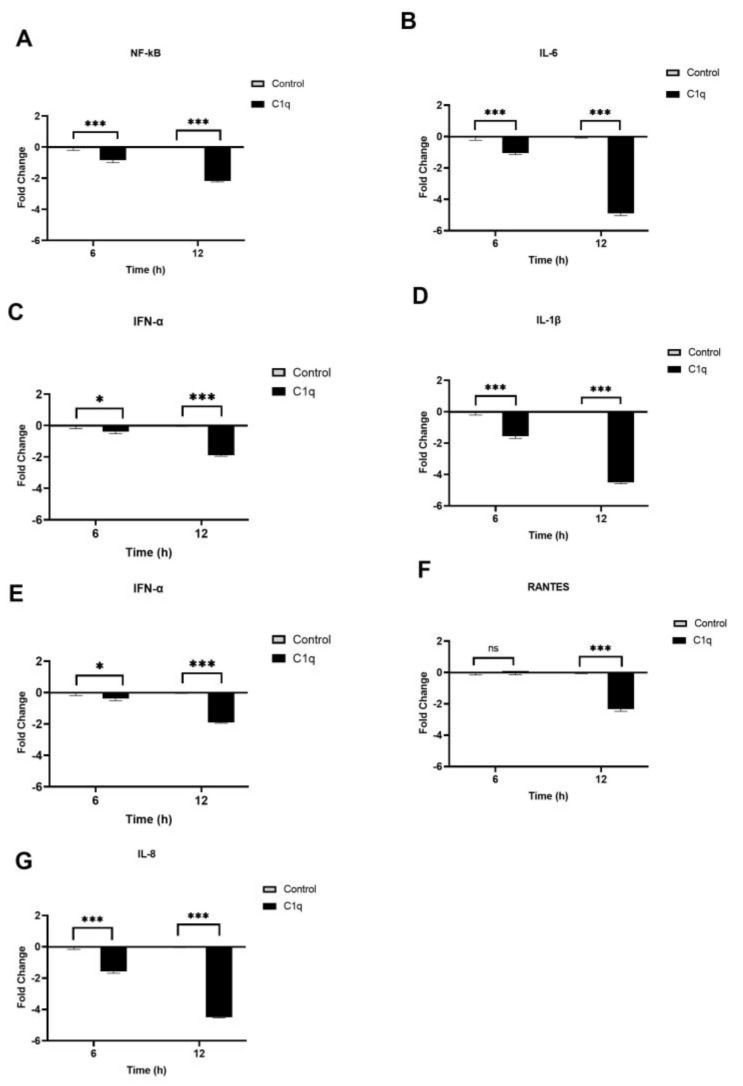
C1q reduces the inflammatory response in SARS-CoV-2 pseudoparticle-challenged A549-hACE2+TMPRSS2 cells. SARS-CoV-2 alphaviral pseudoparticles, pre-incubated with 40 µmol/mL of C1q, were utilised to challenge A549-hACE2+TMPRSS2 cells. The cells were harvested at 6 h and 12 h to measure the mRNA levels of proinflammatory cytokines and chemokines. Cells were lysed, and purified RNA was converted into cDNA. The mRNA levels of NF-κB (**A**), IL-6 (**B**), IFN-α (**C**), IL-1β (**D**), TNF-α (**E**), RANTES (**F**), and IL-8 (**G**) were measured using RT-qPCR; the data were normalised against 18S rRNA expression as a control. The relative expression (RQ) was calculated using A549-hACE2+TMPRSS2 cells challenged with SARS-CoV-2 alphaviral pseudoparticles alone as the calibrator. RQ = 2^−∆∆Ct^ was used to calculate the RQ value. Experiments were carried out in triplicates, and error bars represent ± SEM. Significance was determined using the two-way ANOVA test (* *p <* 0.05, *** *p* < 0.001, ns *p* > 0.05) (*n* = 3).

**Figure 8:**
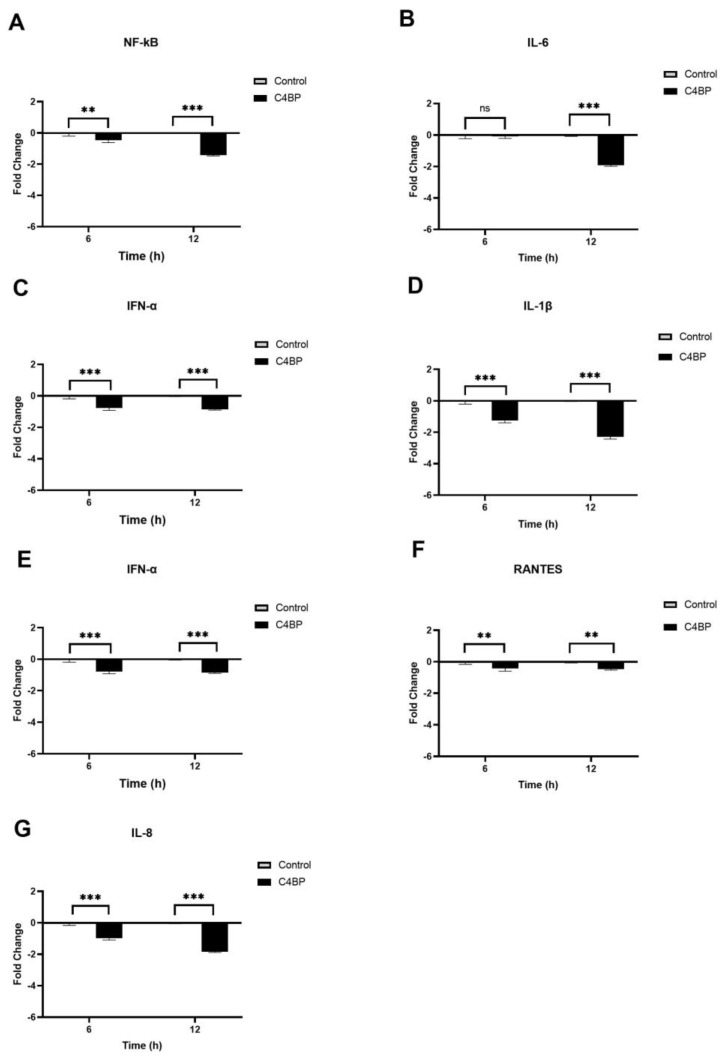
C4BP attenuates the inflammatory response in SARS-CoV-2 pseudotyped viral particle-challenged A549-hACE2+TMPRSS2 cells. The gene expression profile of cytokines and chemokines produced in A549-hACE2 +TMPRSS2 cells challenged with SARS-CoV-2 alphaviral pseudoparticles that were pre-treated with and without C4BP (40 µmol/mL) was examined. Expression levels of NF-κB (**A**), IL-6 (**B**), IFN-α (**C**), IL-1β (**D**), TNF-α (**E**), RANTES (**F**), and IL-8 (**G**) were measured using RT-qPCR at 6 h and 12 h. A549-hACE2+TMPRSS2 cells challenged with SARS-CoV-2 alphaviral pseudoparticles were used as a calibrator to calculate relative quantitation (RQ); RQ = 2^−∆∆Ct^. The experiments were conducted in triplicates, and error bars represent ± SEM. Additionally, 18S rRNA was used as an endogenous control. Significance was established using the two-way ANOVA test (** *p* < 0.01, *** *p* < 0.001, ns *p* > 0.05) (*n* = 3).

We also investigated the modulatory effects of C1q and C4BP on inflammatory gene expression during SARS-CoV-2 infection using RT-qPCR. This was examined by comparing the mRNA levels of proinflammatory cytokines and chemokines, such as interleukin 1 beta (IL-1β), IL-8, tumour necrosis factor-alpha (TNF-α), interferon alpha (IFN-α), nuclear factor kappa B (NF-κB), and RANTES, in treated cells (protein + SARS-CoV-2 alphaviral pseudoparticles + A549-hACE2+TMPRSS2 cells) with their respective controls (SARS-CoV2-alphaviral pseudoparticles + A549-hACE2+ TMPRSS2 cells). The data revealed immune modulation in A549-hACE2+ TMPRSS2 cells by C1q and C4BP (Figure 7 and Figure 8). A549-hACE2+ TMPRSS2 cells, when challenged with SARS-CoV-2 alphaviral pseudoparticles that were pre-incubated with C1q (C1q-treated cells), exhibited lower mRNA levels of IFN-α, IL-6, RANTES, IL-1β, IL-8, and TNF-α, compared to their respective untreated control (Figure 7). C1q caused a reduction in NF-κB gene expression levels at 6 h (−0.8-fold), with a marked effect evident at 12 h (~−2.5-fold) (Figure 7A). At 6 h, C1q-treated cells displayed a decrease in mRNA levels of IL-6 (~−1-fold) (Figure 7B), IFN-α (~−0.3-fold) (Figure 7C), IL-1β (~−1.5-fold) (Figure 7D), and TNF-α (~−1.5-fold) (Figure 7E), compared to their respective controls. Similarly, at 12 h post infection, C1q-treated cells exhibited lower gene expression levels of IL-6 (~ −5-fold) (Figure 7B), IFN-α (~−1.9-fold) (Figure 7C), IL-1β (~−4.5-fold) (Figure 7D), and TNF-α (~−4.4-fold) (Figure 7E). RANTES mRNA levels remained unchanged at 6 h in C1q-treated cells, while at 12 h, a significant reduction was observed (~−2.3-fold) (Figure 7F). C1q-treated cells showed downregulation of IL-8 mRNA levels at 6 h (~−1.5-fold) and 12 h (~−4.7-fold) when compared to their respective controls (Figure 7G).

The immune modulatory effects of SARS-CoV-2 alphaviral pseudoparticles that were pre-treated with C4BP and then challenged against A549-hACE2+TMPRSS2 cells (C4BP- treated cells) were similar to C1q (Figure 8). C4BP-treated cells had reduced mRNA levels of IFN-α, IL-6, RANTES, IL-1β, IL-8, TNF-α, and NF-κB compared to the control cells. At 6 h, C4BP-treated cells showed lower NF-κB gene expression levels (~−0.4-fold) than untreated cells, with further reduction at 12 h (~−1.5-fold) (Figure 8A). No significant change in the mRNA level of IL-6 was observed at 6 h; nevertheless, there was a noticeable downregulation at 12 h (~−1.9-fold) in C4BP-treated cells (Figure 8B). Compared to their respective controls, mRNA levels at 6 h of IFN-α (~−0.8 fold) (Figure 8C), IL-1β (~−1.2-fold) (Figure 8D), TNF-α (−1-fold) (Figure 8E), RANTES (−0.2-fold) (Figure 8F), and IL-8 (~−1-fold) (Figure 8G) were reduced in C4BP-treated cells, whereas at 12 h the mRNA levels of IFN-α, IL-1β, TNF-α, RANTES, and IL-8 were even further downregulated (~−1-fold, ~−2.4-fold, ~−1-fold, ~−0.4-fold, and ~−1.8-fold, respectively). These results suggest that C1q and C4BP attenuate the inflammatory immune response in SARS-CoV-2 infection in a complement activation-independent manner.

## 4. Discussion

The COVID-19 pandemic has led to over 20 million mortalities worldwide [1]. Age, genetics, pre-existing medical conditions, and immune dysregulation increase the risk of developing severe COVID-19 and death [24]. The dysfunction of the complement system is linked to SARS-CoV-2 immunopathogenesis [25]. Various studies have reported that the dysregulation of alternative and classical pathways affects the severity of SARS-CoV-2 infection [10,11]. It has also been shown that the SARS-CoV-2 S protein triggers complement activation [26]. Additionally, lower serum levels of C1q and C4BP in severe COVID-19 have been reported [10,11]. Nevertheless, the immune function of C1q and C4BP against SARS-CoV-2 infection, independent of complement activation, remains unresolved. Therefore, this study investigated the role C1q and C4BP play as immune effector molecules during SARS-CoV-2 infection.

This study reveals that C1q and C4BP can interact directly with SARS-CoV-2 S and RBD glycoproteins. Previous studies have indicated that C1q and C4BP bind directly to viral surface glycoproteins [19,27]. In this study, A549 cells expressing human ACE2 and TMPRSS2 (A549-hACE2+TMPRSS2 cells) along with SARS-CoV-2 lentiviral pseudoparticles were used to assess if C1q and C4BP interfered with SARS-CoV-2 infection at the cellular level. SARS-CoV-2 infection injures alveolar type II cells, causing excessive inflammation and respiratory dysfunction [3]. Alveolar type II cells are essential for innate immunity because they produce and secrete multivalent collectins, SP-A and SP-D, which function as PRRs and opsonin [28]. Alveolar type II cells also secrete proinflammatory mediators and express the human leukocyte antigen–DR isotype, CD80, and CD86 that are required for the antigen presentation [29]. It is thought that alveolar type II cells may function as antigen-presenting cells during viral infections to trigger T cell responses; however, they might be less efficient than dendritic cells [29]. Alveolar type II cell function has been studied using the A549 cells as a physiologically relevant model for the lung epithelium [30].

SARS-CoV-2 has been categorized as a BSL-3 pathogen, severely limiting its use in many facilities due to its highly contagious nature. SARS-CoV-2 pseudotyped viruses can be produced using a surrogate viral core to produce virions showing the SARS-CoV-2 spike protein, with or without the other viral structural proteins such as E, N, and M, allowing them to be used beyond containment level 3 restrictions [23]. In addition, the pseudotyped particles can be used in BSL-2 laboratories due to their single-round infection and replication-deficient features. The use of the pseudotyped particles as a platform to examine sero-surveillance, antigenic characteristics, and entry mechanisms of emerging viruses has been extensively reviewed. Various studies have shown a robust correlation between pseudotyped particle neutralization assays and those for wild-type viruses [31,32].

C1q is the key molecule of the classical pathway; it is produced locally by adherent monocytes and dendritic cells/macrophages [33]. C1q can neutralise IAV in vitro. In addition, C1q interacts with retroviruses involving the globular region of C1q and envelope glycoproteins of several viruses, including gp41 and gp120 of HIV-1, p15E of murine leukaemia virus (MuLV), and gp21 of human T lymphotropic virus (HTLV)-1 (5). C4BP is a major fluid phase inhibitor of the classical and the lectin pathways, which can be produced locally in the lungs by alveolar type II cells [18]. C4BP can facilitate the uptake of adenoviruses by hepatocytes via its interaction with cell surface heparin–sulphate proteoglycans [5]. C4BP also inhibits IAV entry into A549 cells [5]. Thus, the local production of C1q and C4BP may reflect on their significance as effector molecules against microbial invasion.

A cell binding assay verified the effect of C1q, ghA, ghB, ghC, and C4BP on SARS-CoV-2 binding to epithelial-like A549 cells. The binding of complement protein-pre-treated SARS-CoV-2 lentiviral pseudoparticles to A549-hACE2+TMPRSS2 cells was significantly reduced, primarily by C1q and its globular heads compared to the control. C1q and C4BP can limit viral binding of IAV to target host cells independent of complement activation [19,27]. Considering the cell binding results, luciferase reporter gene assays were used to assess whether the effect of C1q, ghA, ghB, ghC, and C4BP on cell binding may impact on viral cell entry. The assay revealed that C1q, ghA, ghB, ghC, or C4BP pre-treated SARS-CoV-2 lentiviral pseudoparticles significantly lowered the viral transduction in A549-hACE2+TMPRSS2 cells as compared to the controls, respectively. The results suggest that C1q and C4BP may be crucial for inhibiting SARS-CoV-2 cell entry into the lung epithelium.

The main factor causing the transition of mild or moderate to severe SARS-CoV-2 infection is inflammation dysregulation [33]. Serum levels of IL-1β, IL-6, IL-8, and TNF-α are elevated in COVID-19 patients and are associated with severe infection [34,35]. The classical pathway is a critical player in the immunopathogenesis of SARS-CoV-2 infection [36]. Additionally, alveolar type II cells exposed to SARS-CoV-2 show raised levels of IL-6, TNF-α, MIP-2, and IL-8 gene expression [37]. Thus, this study employed a qPCR analysis to determine whether C1q and C4BP impacted the expression of proinflammatory genes during SARS-CoV-2 infection in lung epithelial-like A549 cells in a complement-independent manner.

Various studies have demonstrated that the structural proteins of SARS-CoV-2, including S, E, and N, can induce inflammatory responses in the respiratory epithelium [38,39]. Therefore, this study employed SARS-CoV-2 alphaviral pseudoparticles expressing S, E, N, and M proteins as a safe viral model to investigate inflammatory gene expression in a lung epithelium model. This was further confirmed using RT-qPCR, revealing that the SARS-CoV-2 alphaviral pseudoparticles were able to induce an inflammatory response in A549 cells expressing human co-receptors ACE2 and TMPRSS2 (Supplementary Figure S5).

Dysregulation of NF-κB activity has been observed in moderate and severe SARS-CoV-2 infections. NF-κB activity is key to an effective immune response to viral infections [40]. However, the deregulation of NF-κB activities is associated with elevated levels of IL-6, IL-21, IL-1, IL-2, IL-8, MIP-1, MCP1, RANTES, and TNF-α in severe SARS-CoV-2 infection [40]. Therefore, NF-κB inhibition has been suggested as an effective treatment strategy for severe SARS-CoV-2 infection [40]. SARS-CoV-2 alphaviral pseudoparticles, pre-treated with C1q or C4BP, reduced NF-κB gene expression levels in A549-hACE2+TMPRSS2 cells (C1q- or C4BP-treated cells) compared with the control (A549-hACE2+TMPRSS2 cells + SARS-CoV-2 alphaviral pseudoparticles). The NF-κB reporter revealed that C1q- or C4BP-treated cells had lower NF-κB activation levels with respect to the control. These results imply that C1q and C4BP may inhibit an excessive immune response in SARS-CoV-2 infection by decreasing NF-κB activity.

The inflammatory response to viral infection is heavily associated with IL-1β activities [41]. It has been shown that SARS-CoV-2 triggers IL-1β secretion, which can then activate IL-6 and TNF-α [42,43]. SARS-CoV and its ORF3a protein were found to potently induce IL-1β secretion; the mechanism involves the activation of NF-κB and NLRP3 [43]. Elevated serum levels of IL-1β have been linked to IL-6 and TNF-α production through proinflammatory signalling pathways such as NF-κB [44]. In addition, IL-1β promotes the SARS-CoV-2-induced cytokine storm [45]. Patients with severe SARS-CoV-2 infection have shown elevated levels of IL-1β in their peripheral blood and bronchoalveolar lavage fluid (BALF) [46,47]. Targeted IL-1ß treatment has been shown to inhibit SARS-CoV-2-induced cell death [48]. Additionally, IL-1 receptor blocking was an efficient treatment for COVID-19 patients’ respiratory failure, cytokine storm development, and hyperinflammation in the early stages of the infection [49]. In this study, we observed that treatment with C1q or C4BP resulted in decreased levels of IL-1β gene expression. These findings suggest that C1q and C4BP may play a role in reducing the potential of IL-1β to induce excessive inflammation in SARS-CoV-2 infections.

Severe SARS-CoV-2 infection has been associated with higher serum levels of TNF-α [50]. TNF-α is vital for eliminating viral infections [50]. Nevertheless, severe COVID-19 patients exhibit high levels of TNF-α, contributing to lung damage and a poor prognosis [51]. In severe cases of SARS-CoV-2 infection, a combination of anti-TNF-α and anti-IFN-γ therapy was found to minimize tissue damage and mortality [51]. In our study, we have shown that treatment with C1q or C4BP resulted in reduced levels of TNF-α gene expression compared to control cells. These findings suggest that C1q and C4BP may play a role in modulating the immune response by limiting undesirable inflammation and restraining the involvement of TNF-α SARS-CoV-2-mediated immunopathogenesis.

SARS-CoV-2 pneumonia patients had elevated serum levels of IL-6, which is related to the severity and mortality of the disease [52]. IL-6 has been considered as a biomarker in the progression and development of COVID-19 [52]. High levels of IL-6 are linked to a poor prognosis because it promotes inflammation and triggers cytokine storms [52]. Tocilizumab, a monoclonal antibody that targets IL-6 receptors, effectively treats COVID-19 patients susceptible to cytokine storms [52]. In this study, we show that C1q or C4BP l plays a role in reducing IL-6 mRNA levels, thereby preventing the progression of SARS-CoV-2 infection to a severe form of COVID-19.

Increased IFN-type 1 levels (IFN-α and IFN-β) could contribute to various immune mechanisms that promote the severity of SARS-CoV-2 infection [53]. IFN-type 1 is a crucial cytokine in inhibiting viral infection by increasing the expression of interferon-stimulated genes (ISGs) [53]. Early studies have indicated a limited IFN-type 1 response in SARS-CoV-2 infection [53]. Recently, IFN-type 1 has been reported to play a role in the progression of severe SARS-CoV-2 infection [53]. Additionally, a retrospective study found that administering IFN-α in severe SARS-CoV-2 infections increased mortality and slowed recovery, whereas using it in early infection decreased mortality and enhanced recovery [54]. Here, we demonstrated that cells treated with C1q or C4BP had lower levels of IFN-α gene expression than control cells. Thus, C1q and C4BP may be involved in modulating the immune response by preventing undesirable inflammation via restricting the role of IFN-α in developing SARS-CoV-2 immunopathogenesis.

IL-8 is linked to a high level of neutrophil infiltration, respiratory failure, and acute kidney injury in severe SARS-CoV-2 infection [55]. IL-8 is mainly responsible for activating and recruiting neutrophils during inflammation [55]. Neutrophil infiltration is more common in severe COVID-19 patients than those with mild disease [55]. Preventing the onset of severe lung injury is achieved by anti-CXCL-8 therapy [56]. In this study, we found that C1q or C4BP caused a decrease in IL-8 mRNA levels compared to the control group. These findings suggest that C1q and C4BP may play a protective role in mitigating lung damage by inhibiting the effects of IL-8 during SARS-CoV-2 infection.

Finally, compared to healthy controls, patients with mild and severe SARS-CoV-2 infection had higher serum levels of RANTES [57]. RANTES (CCL5) is a robust leucocyte chemoattractant that causes the migration of various immune cells, such as T cells, Natural killer cells, dendritic cells, monocytes, basophils, and eosinophils [58]. High levels of RANTES have been linked to acute renal failure and liver damage in severe COVID-19 [59]. Therefore, early viral clearance and localization of the infection may be improved by targeting RANTES [10]. In our study, we observed a decrease in RANTES gene expression in cells treated with C1q or C4BP compared to the control group. These results indicate that C1q and C4BP may play a regulatory role in limiting viral infection and facilitating viral clearance by modulating RANTES levels in SARS-CoV-2 infection.

Further investigation is needed to understand the interaction of C1q and C4BP with the S protein of various SARS-CoV-2 variants and lineages. This study provides valuable insights into the potential role of C1q or C4BP as immune modulators during COVID-19, but additional experiments utilizing clinical isolates of SARS-CoV-2 from different variants and lineages are necessary to assess the actual infection dynamics. Furthermore, conducting in vivo studies to assess the effects of increased local levels of C1q and C4BP in the lung microenvironment, as well as exploring combination therapies, will be crucial to establish how complement components as soluble pattern recognition receptors can mitigate complications associated with SARS-CoV-2 infection.

**Figure 9:**
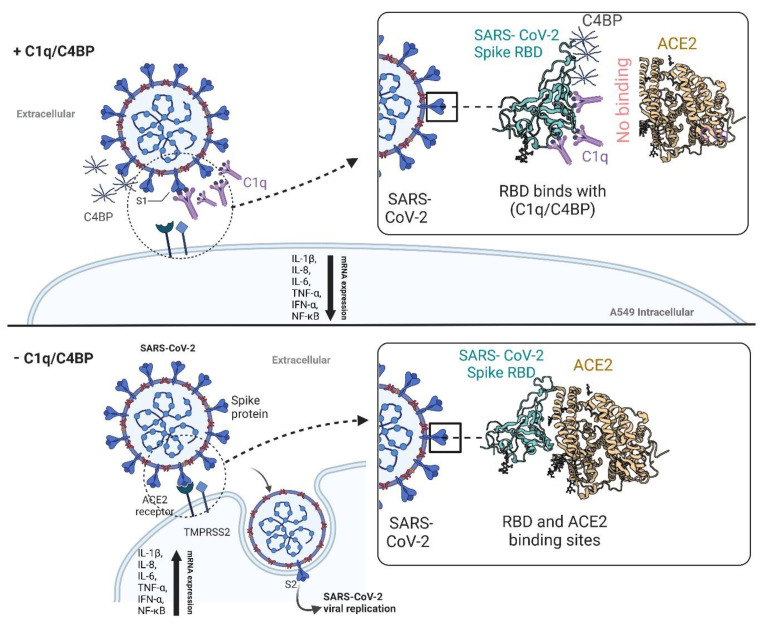
Complement independent attenuation of SARS-CoV-2 infection by C1q and C4BP. The activator of the classical pathway of the complement system, C1q, and the regulatory protein of the classical and lectin pathway of the complement system, C4BP, were found to interact with the receptor-binding domain (RBD) of the SARS-CoV-2 spike protein. This interaction helped reduce SARS-CoV-2 infection in A549 cells expressing human ACE2 and TMPRSS2 by preventing the virus from binding to its cell surface receptors, independent of complement activation. In addition, C1q and C4BP treatment decreased the mRNA levels of proinflammatory cytokines and chemokines (IL-1β, IL-8, IL-6, TNF-α, IFN-α, NF-kB, and RANTES), thereby attenuating infection-associated inflammation. These findings suggest that complement proteins have a novel role as soluble pattern recognition molecules, functioning as one of the first lines of defence against viral infections independent of their complement-related functions. C1q: Complement component 1q; C4BP: Complement component 4 binding protein; RBD: Receptor-binding domain; ACE2: Angiotensin-converting enzyme 2; TMPRSS2: Transmembrane protease serine 2.

In conclusion, this study revealed that C1q and C4BP can interact with the receptor-binding domain (RBD) of the SARS-CoV-2 spike protein in a complement activation-independent manner (Figure 9). This interaction impedes the virus from binding to its cell surface receptors, and thus, reduces SARS-CoV-2 infection in A549 cells that co-express human ACE2 and TMPRSS2. Furthermore, C1q and C4BP decreased the levels of mRNA for proinflammatory cytokines and chemokines, including IL-1β, IL-8, IL-6, TNF-α, IFN-α, NF-κB, and RANTES. Thus, complement proteins may function as soluble pattern recognition molecules, serving as one of the first lines of defence against viral infections, independent of their complement-related functions. This study has helped explain one mechanism of our innate protective arm against SARS-CoV-2 infection.

## Figures and Tables

**Table 1:** Forward and reverse primers used for qRT-PCR assay.

Gene	Forward Primer	Reverse Primer
18S	5′-ATGGCCGTTCTTAGTTGGTG-3′	5′-CGCTGAGCCAGTCAGTGTAG-3′
TNF-α	5′-AGCCCATGTTGTAGCAAACC-3′	5′-TGAGGTACAGGCCCTCTGAT-3′
IL-6	5′-GAAAGCAGCAAGAGGCACT-3	5′-TTTCACCAGGCAAGTCTCCT-3′
IL-8	5′-GTGCAGTTTTTGCCAAGGAG-3′	5′-CACCCAGTTTTCCTTGGGGT-3′
NF-κB	5′-GTATTTCAACCACAGATGGCACT-3′	5′-AACCTTTGCTGGTCCCACAT-3′
RANTES	5′-GCGGGTACCATGAAGATCTCTG-3′	5′-GGGTCAGAATCAAGAAACCCTC-3′
IFN-α	5′-TTTCTCCTGCCTGAAGGACAG-3′	5′-GCTCATGATTTCTGCTCTGACA-3′
IL-1β	5′-GTGCAGTTTTGCCAAGGAG-3′	5′-ACGTTTCGAAGATGACAGGCT-3′

## Data Availability

Not applicable.

## Supplementary Materials

The following supporting information can be downloaded at: https://www.mdpi.com/article/10.3390/v15061269/s1, Figure S1: Characterization of purified complement proteins; Figure S2: Western blot analysis for A549- hACE2 +TMPRSS2 cells expressed hACE2 and TMPRSS2; Figure S3: The viral-cell entry and binding of SARS-CoV-2 lentiviral pseudoparticles into A549 cells; Figure S4: The viral-cell entry and binding of SARS-CoV-2 lentiviral pseudoparticles into A549-hACE2+TMPRSS2 cells; Figure S5: SARS-CoV-2 alphaviral-pseudoparticles induces Inflammatory Response in A549-hACE2 + TMPRSS2 Cells.
