# Supplementary material for: Complement Activation-Independent Attenuation of SARS-CoV-2 Infection by C1q and C4b-Binding Protein
Source: Viruses. 2023 May 29;15(6):1269. doi: 10.3390/v15061269 (PMC10305604; doi:10.3390/v15061269)
Supplement: Supplementary file 1 [file viruses-15-01269-s001.zip › viruses-2373985-supplementary.pdf]

A

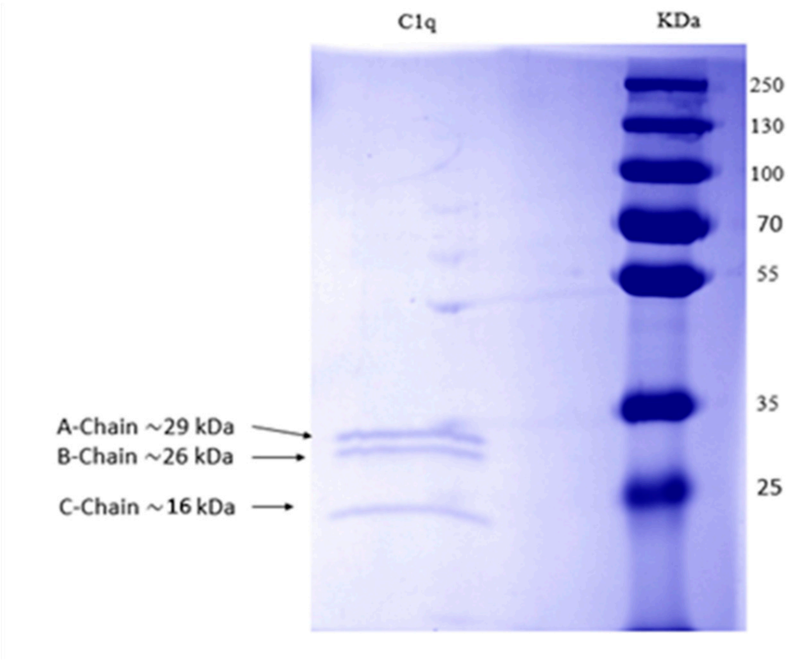
C1q
KDa
250
130
100
70
55
35
25
A-Chain ~29 kDa
B-Chain ~26 kDa
C-Chain ~16 kDa

B

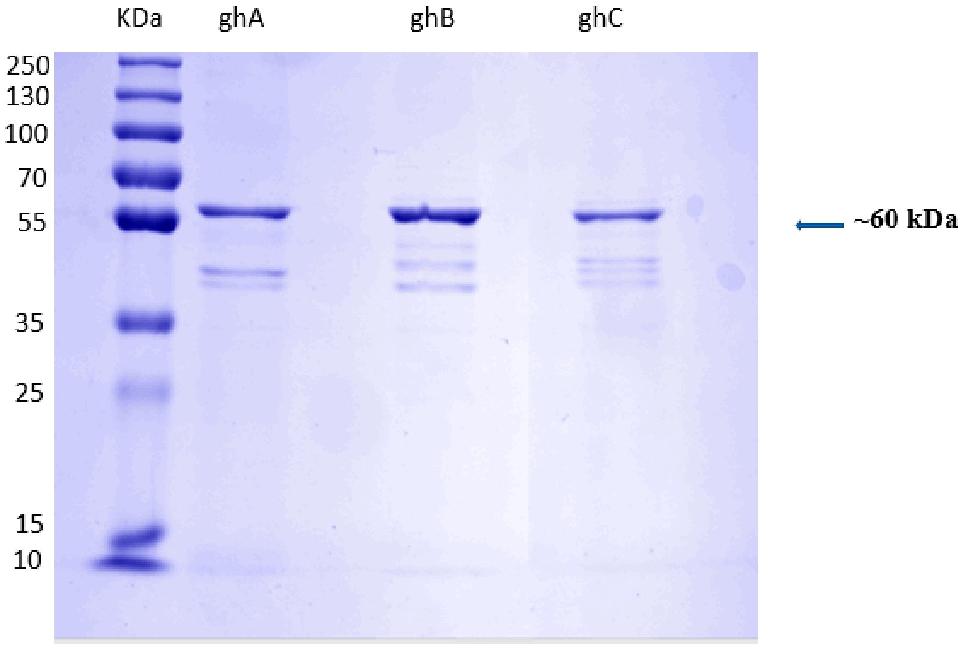
KDa
ghA
ghB
ghC
250
130
100
70
55
35
25
15
10
~60 kDa

C

C4BPα-Chain
~70 kDa

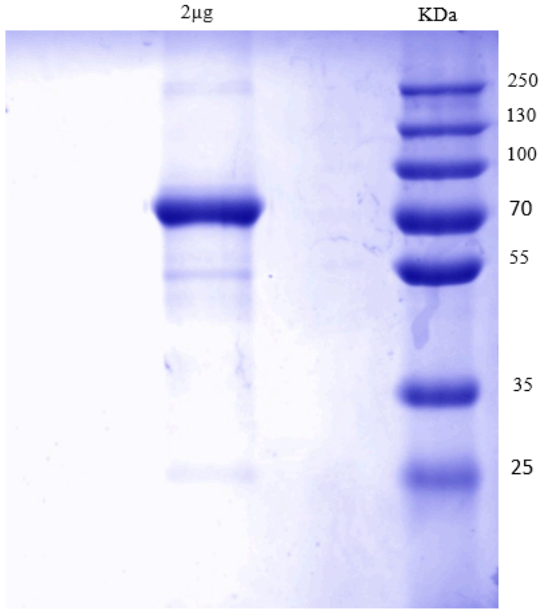

Supplementary Figure 1: Characterization of purified complement proteins. Purified human C1q (A), recombinant globular head ghA, ghB, and ghC modules (B), and C4BP (C) proteins were run via a 12% v/v SDA-PAGE. A protein ladder with a range of 250 to 10 kDa (Thermofisher) was run as a size marker. The protein samples were denatured and reduced, then run for 120 min at 90 V, and stained with Coomassie brilliant blue to reveal protein bands corresponding to C1q [A chain ~29 kDa), B chain (~26 kDa), and C chain (~16 kDa)], recombinant globular heads ghA, ghB, and ghC fused to MBP (~ 60 kDa), and C4BP alpha-chain (~70 kDa).

A

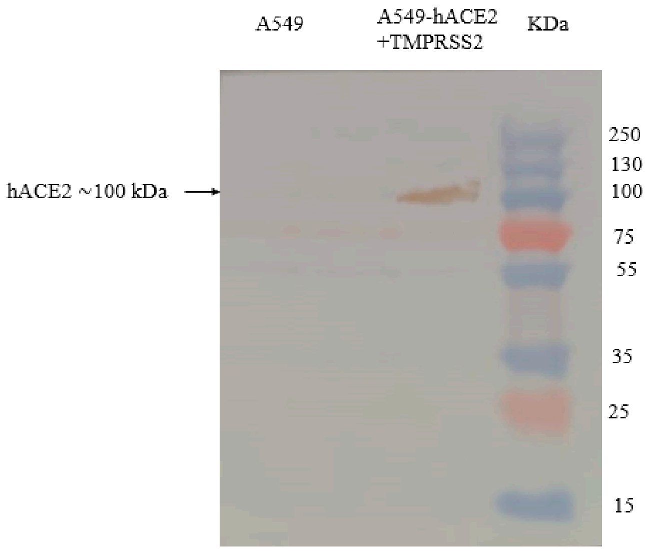

B

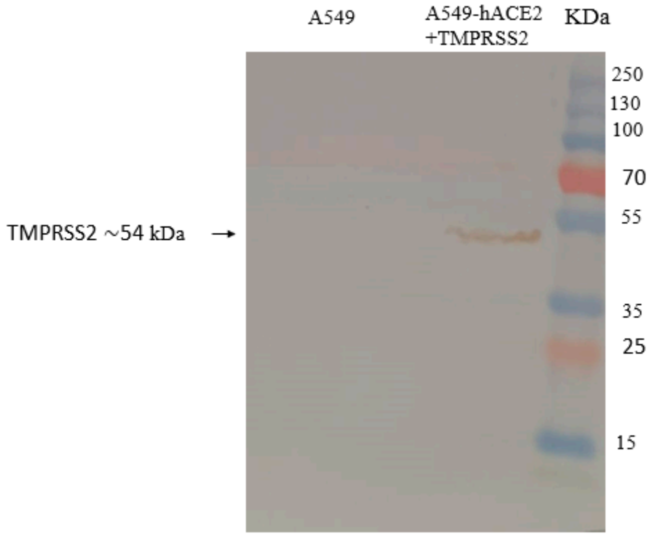

Supplementary Figure 2: Western blot analysis of transfected A549- hACE2 +TMPRSS2 cells showing expression of hACE2 (A) and TMPRSS2 (B). A549 and A549-hACE2+TMPRSS2 cells were harvested and lysed using RIPA buffer. Cell lysates were heated for 10 mins at 95°C, subjected to SDS-PAGE and electro-transferred onto a PVDF membrane. Membranes were blocked in 5% skimmed milk in PBS and incubated for 2h at 4 °C. Next day, the membranes were probed at room temperature for 1h using the corresponding antibodies (1:1,000)- rabbit-anti-human ACE2 and rabbit-anti-human TMPRSS2 polyclonal antibodies, respectively. After developing the colour using 3,3′-diaminobenzidine (DAB) substrate, bands corresponding to the respective proteins, hACE2(~100 kDa), and TMPRSS2 (~54 kDa) were observed.

A

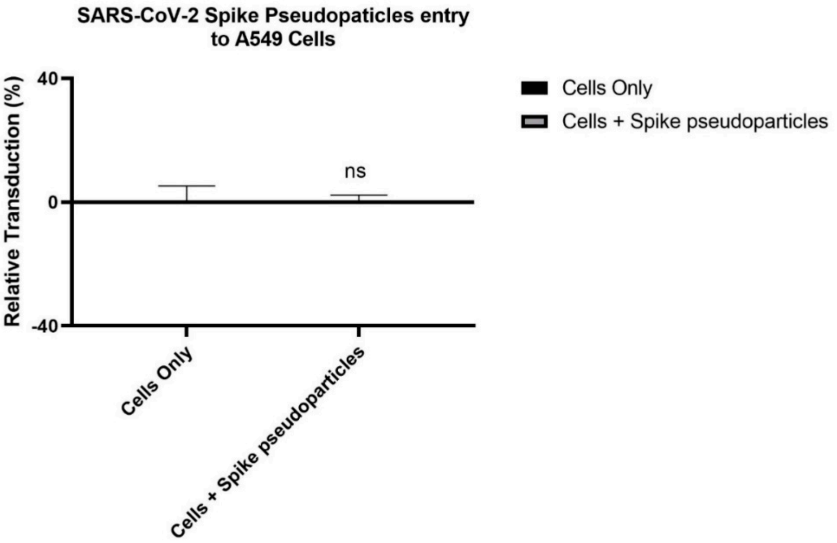

B

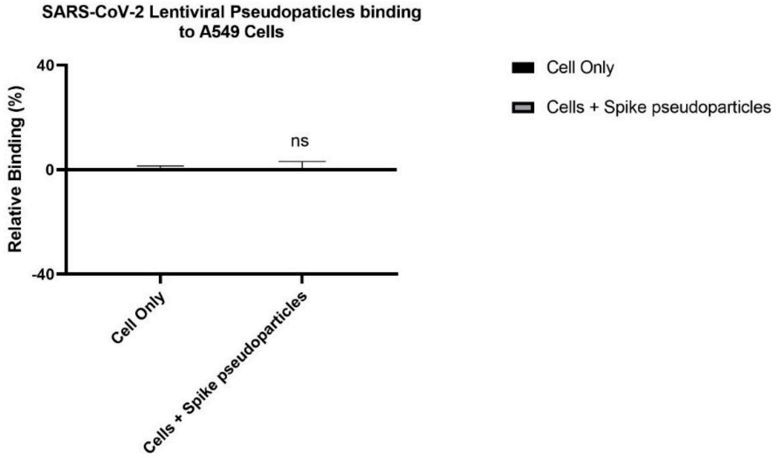

Supplementary Figure 3. Entry of SARS-CoV-2 lentiviral pseudoparticles into A549 cells (A). Luciferase reporter activity of A549 cells, transduced with SARS-CoV-2 lentiviral pseudoparticles, was utilised to assess the lentiviral pseudoparticles' ability to enter the cells. The data obtained were normalised with 0% luciferase activity defined as the mean of the relative luminescence units recorded from the control sample (A549 cells only). Data are shown as the normalized mean of three independent experiments carried out in triplicates ± SEM. Significance was determined using the t- test (ns $p > 0.05$) ($n = 3$). **Binding of SARS-CoV-2 lentiviral pseudoparticles to A549 cells (B).** SARS-CoV-2 lentiviral pseudoparticles were used to challenge A549 cells, and then incubated at 37°C for 2h. The wells were probed with rabbit anti-SARS CoV-2 spike (1:200) polyclonal antibodies after being washed and fixed with 1% v/v paraformaldehyde for 1 min. The data obtained were normalised against 0% fluorescence defined as the mean of the relative fluorescence units recorded from the control sample (A549 cells only). Three independent experiments were carried out in triplicates, and error bars express as ± SEM. Significance was determined using the t-test (ns $p > 0.05$) ($n = 3$).

A

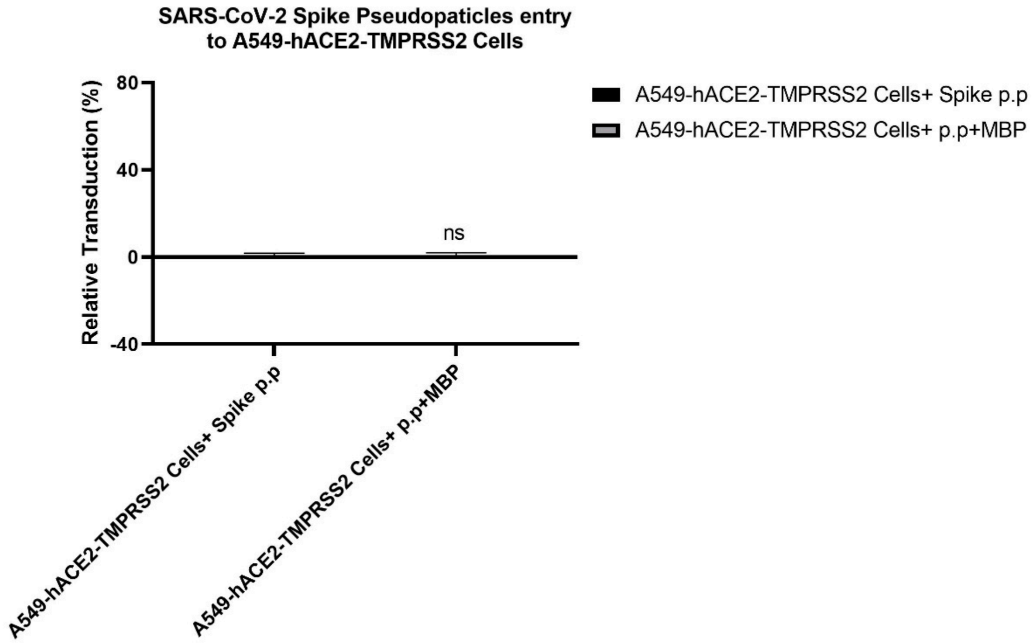
SARS-CoV-2 Spike Pseudopaticles entry
to A549-hACE2-TMPRSS2 Cells
Relative Transduction (%)
80
40
0
-40
ns
A549-hACE2-TMPRSS2 Cells+ Spike p.p
A549-hACE2-TMPRSS2 Cells+ p.p+MBP
A549-hACE2-TMPRSS2 Cells+ Spike p.p
A549-hACE2-TMPRSS2 Cells+ p.p+MBP

B

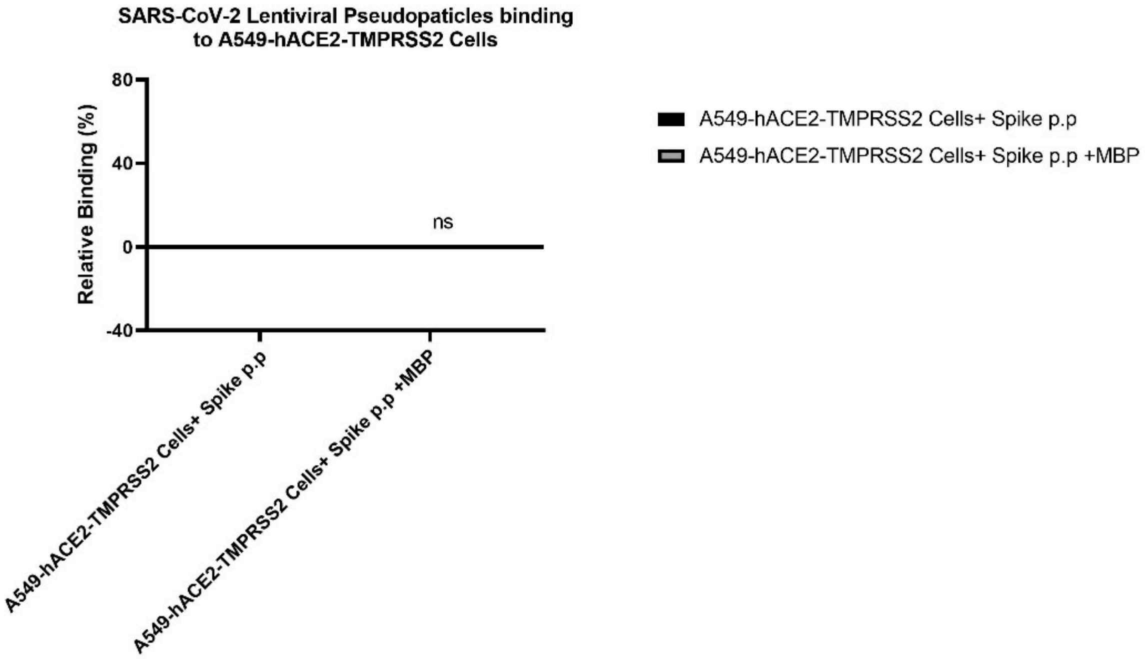
SARS-CoV-2 Lentiviral Pseudopaticles binding
to A549-hACE2-TMPRSS2 Cells
Relative Binding (%)
80
40
0
-40
ns
A549-hACE2-TMPRSS2 Cells+ Spike p.p
A549-hACE2-TMPRSS2 Cells+ Spike p.p +MBP
A549-hACE2-TMPRSS2 Cells+ Spike p.p
A549-hACE2-TMPRSS2 Cells+ Spike p.p +MBP

Supplementary Figure 4. Entry of SARS-CoV-2 lentiviral pseudoparticles into A549-hACE2+TMPRSS2 cells (A). Luciferase reporter activity was measured in A549-hACE2+TMPRSS2 cells that were transduced with SARS-CoV-2 lentiviral pseudoparticles, either pre-treated or untreated with MBP. This assay served to evaluate the ability of the lentiviral pseudoparticles to enter the cells. The obtained data were normalized using 0% luciferase activity, which was defined as the mean of relative luminescence units observed in the control sample (A549-hACE2+TMPRSS2 + SARS-CoV-2 lentiviral pseudoparticles). The results are presented as the normalized mean of three independent experiments, each conducted in triplicates, with the standard error of the mean (SEM) indicated. Statistical significance was determined using the t-test (ns $p > 0.05$) ($n = 3$). **Binding of SARS-CoV-2 lentiviral pseudoparticles to A549-hACE2+TMPRSS2 cells (B).** A549-hACE2+TMPRSS2 cells were challenged with SARS-CoV-2 lentiviral pseudoparticles, either pre-treated or untreated with MBP, and subsequently incubated at 37°C for 2 hours. Following incubation, the wells were washed and fixed with 1% paraformaldehyde for 1 minute, followed by probing with polyclonal rabbit anti-SARS CoV-2-spike antibody (1:200). The obtained data were normalized using 0% fluorescence, which was defined as the mean of relative fluorescence units observed in the control sample (A549-hACE2+TMPRSS2+ SARS-CoV-2 lentiviral pseudoparticles). The experiments were independently conducted three times in triplicates; the error bars represent the standard error of the mean (SEM). Statistical significance was determined using the t-test (ns $p > 0.05$) ($n = 3$).

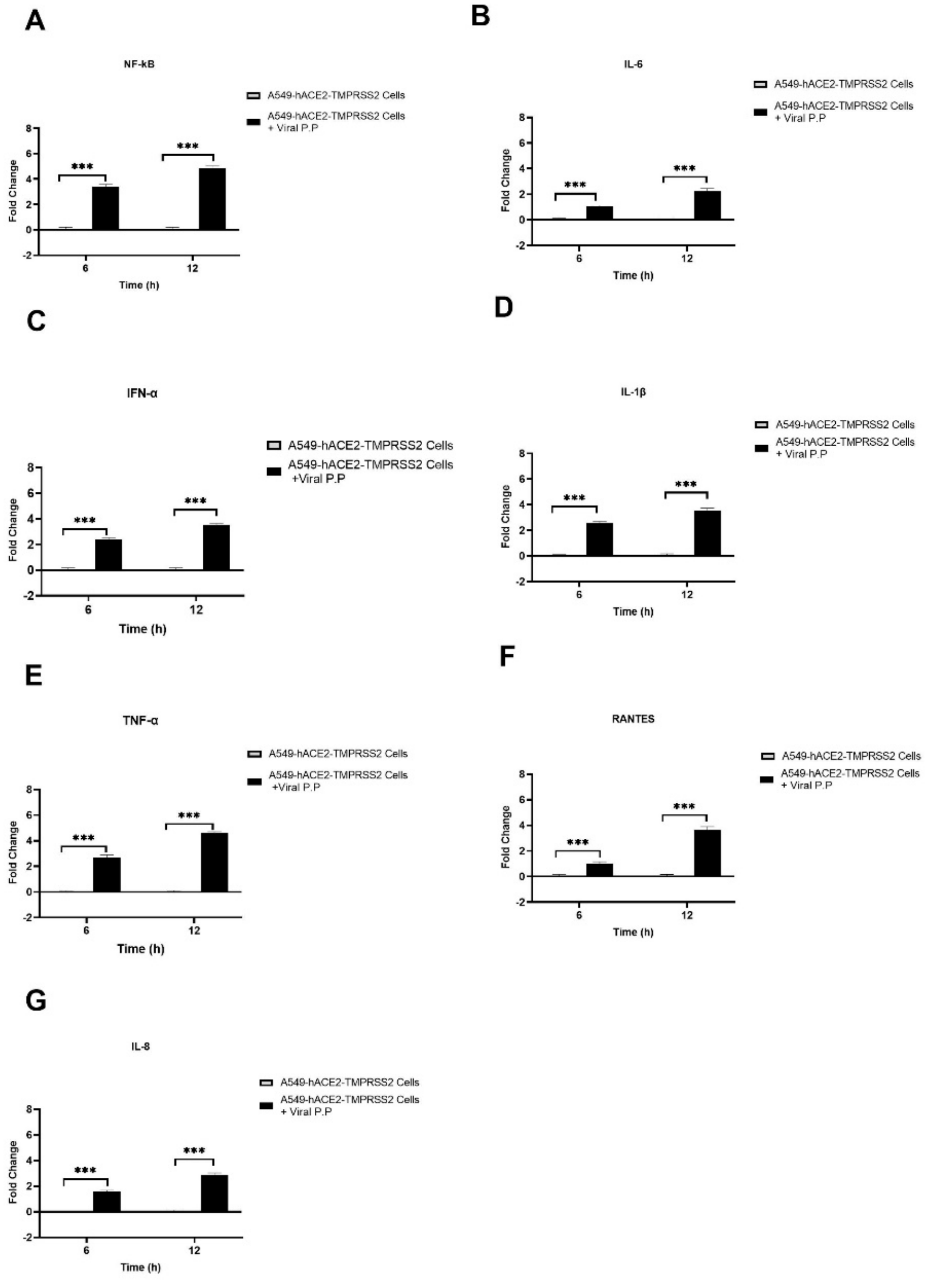

Supplementary Figure 5. SARS-CoV-2 alphaviral-pseudoparticles induce inflammatory response in A549-hACE2 + TMPRSS2 cells. SARS-CoV-2 alphaviral-pseudoparticles were incubated with A549-hACE2+TMPRSS2 cells, and then harvested at 6h and 12h to assess the mRNA levels of proinflammatory cytokines and chemokines. The mRNA levels of NF-κB (A), IL-6 (B), IFN-α (C), IL-1β (D), TNF-α (E), RANTES (F) and IL-8 (G) were measured using RT-qPCR; the data were normalised against 18S rRNA expression as a control. The relative expression (RQ) was calculated using A549-hACE2+TMPRSS2 cells only as the calibrator. RQ = $2^{-\Delta\Delta Ct}$ was used to calculate the RQ value. SARS-CoV-2 alphaviral-pseudoparticles induced pro-inflammatory response in A549-hACE2+TMPRSS2 cells. Experiments were carried out in triplicates, and error bars represent ± SEM. Significance was determined using the two-way ANOVA test (***$p < 0.001$) ($n = 3$).
